# Drawing a line from CO_2_ emissions to health—evaluation of medical students’ knowledge and attitudes towards climate change and health following a novel serious game: a mixed-methods study

**DOI:** 10.1186/s12909-024-05619-4

**Published:** 2024-06-05

**Authors:** Merel Stevens, Adriana Israel, Anouk Nusselder, Juliette C. Mattijsen, Feng Chen, Vicki Erasmus, Ed van Beeck, Suzie Otto

**Affiliations:** 1 Department of Public Health, Erasmus University Medical Center, P.O. Box 2040, 3000 CA Rotterdam, the Netherlands; 2 IFMSA, Joan Muyskenweg 38, P.O. Box 8628, The Netherlands 1114 AN Amsterdam,; 3 Dutch Green Health Alliance (GZA), Botersloot 177, 3011 HE Rotterdam, the Netherlands

**Keywords:** Planetary health, Serious game, Climate change, Medical education, Health

## Abstract

**Background:**

Education is urgently needed to equip medical students with knowledge, values and skills to promote planetary health. However, the current literature offers little insight into evidence-based approaches and best practices. In response to this pressing need, a novel serious game was introduced into the medical curriculum at Erasmus Medical Center in 2023. The aim of this study was to evaluate the knowledge and attitudes of medical students after they had played a serious game that addresses climate change and health.

**Methods:**

In accordance with a mixed-methods design, quantitative data were collected using pre- and post-intervention surveys. Differences were assessed using the Wilcoxon signed rank test. Focus group discussions were held after the game and thematically analysed.

**Results:**

One hundred forty-five students (38.6% of the entire cohort) played the game, of which 59 students completed the pre- and post-intervention surveys. After the game, self-reported knowledge increased. Regarding objective knowledge, an increase in the proportion of students who answered one of the two questions correctly was observed, while the proportion of correct responses decreased for the other question. Student’s responses to two out of five attitude questions were significantly more positive. The proportion of students who recognized the importance of climate change education, to inform patients and society about the health impacts of climate change, increased. Moreover, survey results indicated a significant increase in climate worry subsequent to the game. Eleven students participated in the focus group discussions. Thematic analysis highlighted participants’ reflections on the roles and responsibilities in climate change and health, along with their realisation of the tools for action that climate and health co-benefits provide. Another significant aspect was the importance participants placed on learning alongside peers with diverse attitudes. Additionally, participants appreciated the tangible overview of climate change and health provided by the serious game.

**Conclusions:**

Our novel serious game addressed an important gap in the medical curriculum. The game can enable medical students to cultivate the necessary knowledge and attitudes to promote health in times of a climate crisis. The accompanying climate worry needs attention through the empowerment of students’ agency to foster change.

**Supplementary Information:**

The online version contains supplementary material available at 10.1186/s12909-024-05619-4.

## Introduction

The effects of climate change on human health are regarded as ‘the biggest public health threat of the century’ [1]. Rising temperatures present a substantial risk to human health, food security, water supply and livelihoods [1, 2]. Heat-related mortality, cardiovascular disease and altered distribution patterns of infectious diseases merely scratch the surface of the myriad health impacts of climate change [1, 2]. The healthcare sector, with its objective of safeguarding and promoting health, is a significant emitter of greenhouse gases, accounting for approximately 4.6% of global net emissions. [2] Health systems require immediate transformation and resilience-building against the consequences of climate change and other planetary crises, such as air pollution and biodiversity loss. The current and future health workforce plays a crucial role in this endeavour.

Climate change is a critical component of an emerging discipline called planetary health. Planetary health is defined as the intersectionality between the health of the environment and the health of human individuals, while acknowledging the anthropogenic nature of environmental disruption [3]. The Association for Medical Education in Europe (AMEE) asked for the integration of planetary health education into medical curricula. According to their call, the primary objective of education on this topic is to build students’ knowledge and attitudes towards safeguarding the health of both humans and the planet [4]. Climate change is defined as one of the nine planetary boundaries that together describe the environmental limits within which humanity can operate safely to avoid irreversible changes to the planet’s ecosystems. Other examples of planetary boundaries include biodiversity loss and freshwater use, which are equally important for maintaining a stable and hospitable environment for life on Earth. Staying within these boundaries ensures the long-term health and resilience of both humans and the planet [5]. Although crossing the planetary boundary of climate change has far-reaching consequences for human health, only 15% of medical schools globally included elements of climate change and health in their curriculum [6]. A 2021 Dutch Association of Medical Students survey revealed that, out of 3489 respondents, 83% expressed a desire to learn more about the intersection between climate change, health and sustainability in healthcare in their curriculum [7].

In light of the clear urgency of climate change and health education in medical curricula, the current literature is abundant with calls for action and educational frameworks; however, these frameworks offer little insight into evidence-based approaches and best practices [8–10]. To answer this call for change, the Erasmus University Medical Center (Erasmus MC) in Rotterdam, the Netherlands implemented a collaborative serious game that teaches the interlinkages between climate change and human health. Serious games are designed for purposes beyond entertainment, often with educational objectives, and have been recognized as a potentially meaningful tool for enhancing medical education [11, 12]. In this case, engaging and game-like elements facilitate learning about climate change and health, and stimulate students to reflect on their role in advocating for climate-resilient health systems.

In 2023, the serious game was embedded in a public health course for third-year bachelor medical students at Erasmus MC for the first time. The primary objective of this study was to evaluate students’ knowledge and attitudes towards climate change and health following the serious game. The secondary objective was to identify opportunities and strategies for improving the game.

## Methods

### Study design

A mixed-methods study design was adopted to evaluate students’ knowledge and attitudes towards climate change and health following a serious game. Quantitative data were collected using pre- and post-intervention questionnaires. Qualitative data were gathered from focus group sessions held with a subsample of participants after the game.

### Setting and participants

The study was conducted at Erasmus MC in Rotterdam, The Netherlands. Medical education at Erasmus MC consists of a three-year bachelor’s phase followed by a three-year Master’s phase. The study was carried out during a four-week introductory course on public health for third-year bachelor students in February 2023. The students had not received prior formal education on planetary health or climate change and health. A 45-min plenary introductory lecture on planetary health was included in the course (JM, AN, FC). The lecture provided an introduction to planetary health, a brief overview of the health impacts of climate change and its effects on health disparities, and discussed the concepts of mitigation, adaptation and health co-benefits, applied to the health sector. The lecture was followed by 14 small-group sessions occurring one to four days later. Attendance at the lecture and small-group session was voluntary, but the content was included in the mandatory course examination. The small-group sessions were scheduled for the entire cohort and each session allowed 20–24 students to participate in a serious game. Students were eligible for participation in the study if they were third-year medical students at Erasmus MC.

### Serious game

The content of the serious game was based on the scientific findings of the Lancet Countdown 2022 report [13]. The aim of the game is to teach students about the complex relationship between climate change and health, and about the role of the health sector in climate mitigation and adaptation. After participation in the game, the student will be able to:

- explain the various health impacts of climate change, explain how they occur, and reason how and why different populations are more or less vulnerable to them (Bloom’s taxonomy: remember and understand);
- explain how climate mitigation and adaptation (in healthcare and beyond) and their health co-benefits can be used for health promotion (Bloom’s taxonomy: understand);
- reflect on one’s own role in the public climate debate and in sustainability in healthcare (Bloom’s taxonomy: apply) [14].

**Fig. 1:**
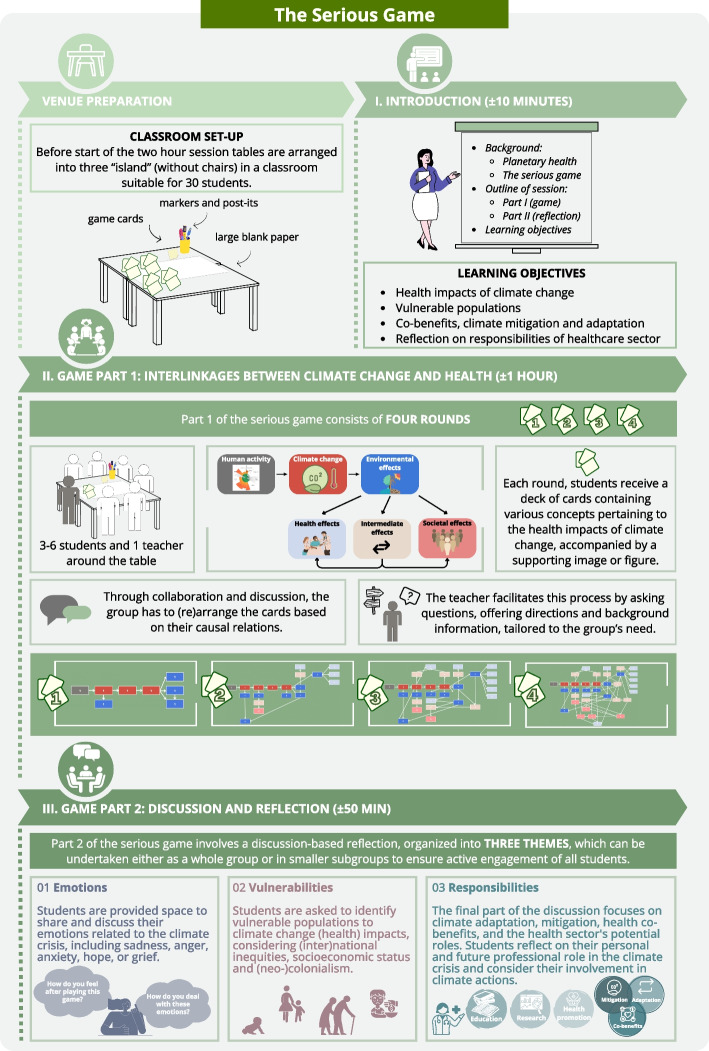
Overview of the serious game

An infographic presenting an overview of the serious game, including the venue setup and aims and learning objectives is presented in Fig. 1.

The two-hour sessions contained the following three components:

1. An introduction—in which the teachers (2-3) introduced themselves, the theme, outline and learning objectives;
2. Part 1 of the game – in which the interlinkages between climate change and health were discussed in smaller subgroups (3–6 students). The students were allocated to small subgroups, either by assignment of the teacher or according to their own preferences. The game was played in four rounds. The card deck of round 1 included mostly the steps in which human activity leads to climate change through several environmental changes. The cards of round 2 to 4 gradually elaborated on this by adding intermediate steps and their ultimate health and societal impacts to the overview. From round 2 to 4, the focus shifted gradually from individual health impacts such as cardiovascular disease (round 2) to societal impacts such as migration and displacement (round 4). 
  The teacher explained the planetary boundaries concept and the focus of the game on the planetary boundary of climate change in relation to health during the first round. During each round of Part 1, students were handed a deck of cards that named different concepts related to the health impacts of climate change, supported by an image or figure. Through collaboration and discussion, the group had to rearrange these cards based on their causal relations. During and between rounds, the teachers supported this process by asking additional questions, offering directions and background information, tailored to the group’s needs;
3. Part 2 of the game – a group discussion and reflection on personal and professional responsibilities (either with the entire group or in the same smaller subgroups). The group discussion was structured to include the following themes:
  *Emotions*: space was provided for the students to share and discuss the emotions they had related to the climate crisis, such as sadness, anger, anxiety, hope or grief and how they could deal with them;
  *Vulnerabilities*: students were asked which populations are particularly vulnerable to the (health) impacts of climate change and why. They discussed inequities within national and international contexts, their relation to socioeconomic status and (neo-)colonialism, and the impacts on specific vulnerable populations.
  *Responsibilities*: the final part of the discussion focused on personal and professional responsibilities. The definitions of climate adaptation, mitigation and health co-benefits, and possible roles for health professionals within each of these domains were discussed. For instance, the benefits of advising a patient to switch to a plant-based diet for the patient’s health and the environment were demonstrated. Students were stimulated to reflect on their own role in the climate crisis, and possible climate actions they can take in their personal and future professional life.  

The sessions were facilitated by a pool of 13 teachers (final-year medical students, public health researchers) with knowledge of the topic. They attended a three-hour teacher training session beforehand and were provided a teacher manual. During the training, they played the game and received practical tips and instructions for facilitation. Special emphasis was placed on facilitating the reflection around emotions, prompting teachers to share their own feelings about the climate crisis. They were instructed to actively encourage students to voice all kinds of emotions after playing the game.

### Quantitative data collection and analysis

All third-year Bachelor students who attended the small-group session (*n* = 145; 38.6% of the entire cohort) were invited to participate in this study. The students were asked to scan a QR-code at the start of the session. They were then directed towards an online version of the participant information leaflet, that informed students about the research objective, (dis)advantages of participation and handling and storage of data, and an informed consent form in LimeSurvey [15]. Participation in the study was not required to attend the session. After informed consent was provided voluntarily, the students were asked to complete the pre-intervention questionnaire (see Additional file 1) in LimeSurvey. At the end of the small-group session, directly after playing the game, the students were asked again to complete the post-intervention questionnaire (see Additional file 1) in LimeSurvey.

The pre-intervention questionnaire was divided into three parts:(1) demographic information, (2) knowledge about climate change and health and (3) attitudes towards climate change and health and its importance for medical students/doctors. The post-intervention questionnaire was the same, except for the substitution of demographic information with evaluation questions. In addition, respondents were asked whether they attended the introductory lecture and were willing to participate in a focus group discussion for further evaluation of the serious game.

The questions were designed by the research team, in line with the core objectives relevant to planetary health education in the medical curriculum at Erasmus MC, and informed by similar studies [16, 17, 18]. The knowledge part consisted of three self-reported knowledge questions and two exam questions to objectively evaluate respondent knowledge. The majority of the attitude and evaluation questions and the self-reported knowledge questions used 5-point Likert scales (*strongly agree-strongly disagree*). The attitude part included two existing European Social Survey questions on climate change worry and belief, which were equally measured on a 5-point Likert scale [19].

#### Statistical analysis

Survey questions with 5–point Likert scales were converted into numerical values and clustered into three categories: Strongly agree/agree (1-2), neutral (3), and disagree/strongly disagree (4-5). The examination questions comprised four statements, two of which were true, and were categorized as follows: 0 or 1 statement, 2 statements and 3 or 4 statements correctly indicated as true or false.

Descriptive statistics were used to characterize the sample, and are summarised by the number of cases (*n*) and percentages (%) for categorical variables and mean and standard deviation (SD) for continuous variables. Differences between pre- and post-intervention responses regarding knowledge and attitudes were assessed using the Wilcoxon signed rank test. Bonferroni correction for multiple testing was applied to determine the level of significance (0.05 (original *p*-value)/12 (number of tests performed) = *p* < 0.004). Analyses were performed using IBM SPSS Statistics for Windows, version 28 [20].

#### Qualitative data collection and analysis

Sampling and number of focus groups were based on the availability and willingness of students to participate. Students were invited to participate in the focus groups in the pre-intervention survey and announcements were posted on the online learning management system and in the cohort’s WhatsApp group*.* Two focus group discussions of 90 min each were conducted, one month post-intervention, in a meeting room at Erasmus MC. The sessions were facilitated by MS and AI, and supervised by VE. The facilitators alternated between moderators and note-takers. AI had met two of the participants prior in her role as a teacher during other education. The facilitators did not teach the serious game to any of the focus group participants. MS and AI maintained a neutral stance while encouraging participants to express diverse opinions.

Eleven students consented for participation in the focus group discussions. The first focus group consisted of six students (three females); five students attended the second focus group (four females). Participants received an information leaflet and opportunity to ask questions. The sessions were audio recorded, after verbal consent was obtained.

A set of unbiased, open-ended questions and probes was developed by the entire research team (see Additional file 2). While the quantitative study focused on the effect of the serious game on students’ knowledge and attitudes towards climate change and health, the primary objective of the qualitative study was to explore the underlying mechanisms that led to the observed outcomes. To support this objective, the attitudes of the focus group participants towards climate change in general and in relationship with human health were explored prior to asking the main questions about their experiences with the serious game. In addition, the perceived strengths and opportunities for improvement of the serious game were discussed.

#### Qualitative analysis

The audio recordings of the focus group discussions were transcribed verbatim (MS, AI) and de-identified. The six-stage thematic analysis framework by Braun & Clarke was adopted, which includes familiarization with the data, inductive generation of initial codes, searching for themes, reviewing themes, defining and naming themes, and writing the final report [21]. Using NVivo12 software, the initial coding of transcripts was performed independently by MS and AI and their analyses were compared [22]. Finally, the codes were grouped into themes. Themes were reviewed by three authors (MS, AI, VE) in relation to the generated pool of codes and the entire dataset. Finally, definitions and names were generated for each theme. The themes are presented in the text with illustrative quotations.

### Ethics

This study was deemed exempt from additional review according to the Dutch Medical Research with Human Subjects Law by the Erasmus MC Ethical Review Committee (ref. no. MEC-2023–0037).

## Results

### Study Sample

In February 2023, 14 small-group sessions took place, and the game was played by a total of 145 students (38.6% of the entire cohort). A total of 76 students (52.4% of invited students) completed at least the pre- or post-intervention questionnaire. Students who completed only the pre- or only the post-intervention questionnaire (*n* = 17) were excluded from the analysis. The final sample for assessment of knowledge and attitudes therefore consisted of 59 students (40.7% of invited students; 14.6% of the entire cohort).

The mean age of the final sample was 21 years; 46 respondents were female (78%),12 were male (20.3%) and one participant chose not to disclose their gender. Among the participants, 39% attended the introductory lecture prior to the serious game in person, and 40.7% of the participants attended the introductory lecture online.

The serious game was evaluated via the post-intervention questionnaire. More than 90% of the respondents were positive (agree/strongly agree) about the content and methodology of the serious game. It was graded an average of 7.9 out of 10(SD 0.9).

The study findings are outlined below, commencing with knowledge, followed by attitudes and, finally, strategies for improvement. In each section, the quantitative results are presented first, followed by the themes generated from the qualitative findings. An overview of the themes can be found in Table 1. Additional file 3 presents all quantitative results.

**Table 1:** Overview of the themes, developed from thematic analysis

**Related to**	**Themes**
Knowledge	Overview of complex interlinkages between climate change and health
	The least responsible suffer the greatest consequences
	Instrument to acquire an in-depth level of knowledge
Attitude	The climate dilemma: Personal contributions help to get a handle on reality, but seem anything but efficient
	The serious game fuels climate worry
	Tangible overview of playing cards on the table releases wow-effect
	Learning with and from different opinions and attitudes
	Forced to reflect on their own role as a medical student and future MD
	Realising that co-benefits of climate action for health provide tools for action
Strategies for improvement	Uniformity of teachers’ approach
	Final exchange between groups to stimulate peer-learning
	Integration of climate change and health education across the curriculum

### Knowledge

#### Quantitative results

**Fig. 2:**
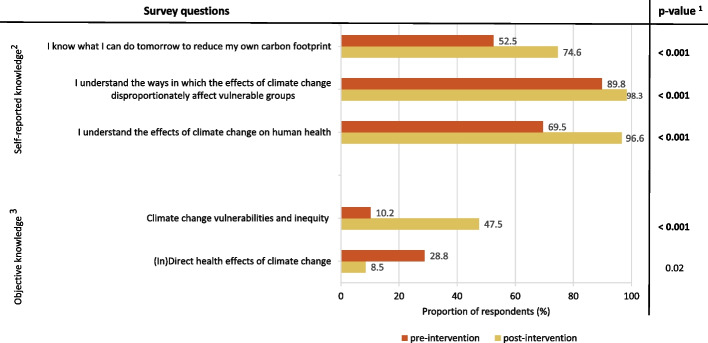
Knowledge about climate change and health before and after the serious game. ^1^Wilcoxon signed-rank test on a 3-point scale: Strongly agree/agree-neutral-disagree/strongly disagree, or 0/1 correct statement-2 correct statements-3/4 correct statements. The bold p-values denote statistical significance (Bonferroni *p* < 0.004).^2^ Self-reported knowledge: The proportion of agree/strongly agree is reported. ^3^ Objective knowledge: The proportion of 3 or 4 correct answers is reported

Figure 2 summarises the pre-and post-intervention results regarding knowledge about climate change and health. The proportion of students who agreed/strongly agreed with the self-reported knowledge statements significantly increased (*p* < 0.001). In terms of objective knowledge, a significantly greater score was observed in response to the question addressing climate change vulnerability and inequity (*p* < 0.001). In contrast, the question about (in)direct health effects of climate change was scored lower in the post-intervention questionnaire, but this finding lost significance after Bonferroni correction (*p* = 0.02). When stratified by gender, the aforementioned findings on knowledge remained consistent with the overall sample.

#### Qualitative results

##### Overview of complex interlinkages between climate change and health

Focus group participants expressed that participation in the serious game enhanced their understanding of the intricate relationship between climate change and human health.

Participants perceived active and collaborative engagement with placing the cards on the table and establishing connections between them as helpful for grasping ‘*the bigger picture*’, the complexity of the problem and its far-reaching consequences for health at both the individual and societal level. Elucidating and discussing the hierarchy and order of the cards displayed supported students’ comprehension of the impact of climate change. They described that playing the game in small groups forced them to actively participate and critically reflect on their own understandings through discussion with their peers.

*[in response to the request to order the cards]* ‘…*that you were to organize the health consequences by yourself. And also neatly place them above and below each other. That provided an overview for me, so that you leave with a sort of mental structure. During the group discussion, you would reconsider, ‘Yes, this card fits here or much earlier’, and then you start to see more connections in your mind.’ – focus group 1, Isaac (male).*

##### The least responsible suffer the greatest consequences

In one of the focus groups, participants indicated that the serious game taught them the uneven distribution of the climate change burden across countries and populations. The role of inequality was highlighted as a previously unknown yet significant aspect of the problem: ‘*It’s not just a kind of natural phenomenon, […] but it’s also truly an unequal thing […]. That it’s also a social issue, alongside being purely geological […].’ – focus group 1, Frank (male)* Participants expressed a sense of moral responsibility as citizens of a high-income country to limit their carbon footprint.

##### Instrument to acquire an in-depth level of knowledge

According to the participants, the planetary health introductory lecture prior to the serious game offered a basic level of knowledge. This was perceived as necessary to be able to actively contribute during the small-group session. The different levels of knowledge among players in the game stimulated them to share knowledge with one another. The aforementioned factors, along with the presence of a teacher who could clarify uncertainties, helped participants to deepen their comprehension of the topic.

*“To hold a group discussion in this way while playing the game, you find out what the group thinks is important and on which topics the group lacks sufficient knowledge. Then you talk about those points together and everyone puts forward new insights. And yes, that works so well that the information sticks with you.” – focus group 1, Hannah (female).*

### Attitude

#### Quantitative results

**Fig. 3:**
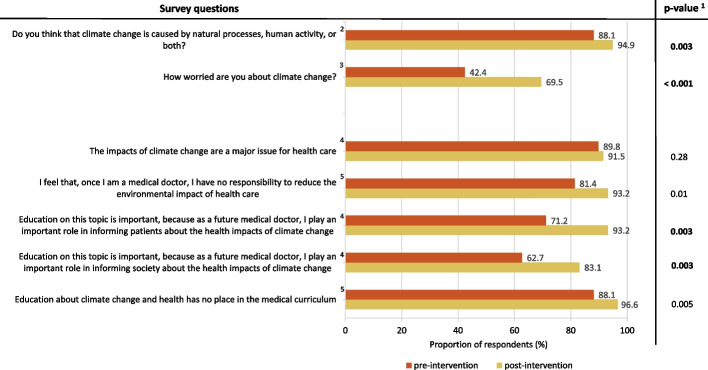
Attitudes towards climate change and health before and after the serious game. ^1^ Wilcoxon signed-rank test on a 3-point scale: Entirely/mainly by natural processes—equally by natural processes and human activity-mainly/entirely by human activity; not at all worried/not very worried-somewhat worried-very worried/extremely worried, or strongly agree/agree-neutral-disagree/strongly disagree. The bold p-values denote statistical significance (Bonferroni *p* < 0.004). ^2^ Proportion of mainly/entirely by human activity is reported. ^3^ Proportion of very/extremely worried is reported. ^4^ Proportion of agree/strongly agree is reported. ^5^ Proportion of disagree/strongly disagree is reported

Figure 3 summarises the pre-and post-intervention results regarding attitudes towards climate change and health. Students reported being more worried about climate change after playing the game (from 42.4% to 69.5%; *p* < 0.001). Similarly, a significantly greater proportion of students acknowledged the anthropogenic nature of climate change after the game (from 88.1% to 94.9%; *p* = 0.003). Regarding the importance of climate change for healthcare and their personal roles and responsibilities, a significant increase in the proportion of students who agreed/strongly agreed with the statements ‘education on this topic is important because as a future medical doctor, I play an important role in informing patients/society about the health impacts of climate change’ was noted (Patients: From 71.2% to 93.2%; *p* = 0.003/Society: From 62.7% to 83.1%; *p* = 0.003). When stratified by gender, the results regarding attitude remained consistent with the overall sample.

#### Qualitative results

##### The climate dilemma: Personal contributions help to get a handle on reality, but seem anything but efficient

Participants shared a sense of being caught in a climate dilemma. On the one hand, they indicated that climate change plays a role in their daily lives to various extents. Making small adjustments as individuals, as long as they are considered reasonable and affordable, seemed to provide them with a feeling of control over the situation. However, they expressed scepticism regarding the efficacy of their own behavioural changes, as they deemed their personal efforts of insignificant contribution to the Paris Agreement. It was repeatedly emphasized that responsibility rests with politicians and governments.

*‘I find it a bit difficult; on one hand, I think it should come from the bottom-up, that people themselves are willing to, and on the other hand […] if it’s imposed by policy, it makes such a bigger difference than if I were to do it alone.’ – focus group 1, Jake (male).*

##### The serious game fuels climate worry

The focus group participants expressed serious concerns regarding climate change and its impact. They articulated feelings of grief and hopelessness: ‘*When I think about it, it does make me a bit sad. It’s kind of a doomsday scenario that is being portrayed.’ – focus group 2, Eliza (female). *The game made them realize that the consequences of climate change can no longer be fully prevented. To some extent, this resulted in acceptance of reality but also evoked a sense of despair. They expressed a lack of trust in current global actions, and said that reaching climate change mitigation targets seems impossible. Even though participants expressed to think about ways of sustainable living for themselves, this increased climate worry did not appear to spark immediate action in the weeks between the lesson and the focus groups.

##### Tangible overview of playing cards on the table releases wow-effect

There was a strong consensus among participants that by seeing the numerous playing cards organized on the table, the realization dawned on them that climate change is a comprehensive societal problem. The overview of playing cards on the table was ‘*an eye-opener that so many things are involved’ – focus group 2, Carmen (female)*. This raised their awareness of the importance and urgency of climate change, as well as of the health effects that are already occurring today.

##### Learning with and from different opinions and attitudes

The participants primarily played the game with fellow students who had a similar positive attitude towards the topic. However, they mentioned that sharing different attitudes among students allows for new insights and encourages solid explanation and justification of one’s own perspective. It was also mentioned that students who deny the problem could hinder the learning process.

‘*I think that it’s always valuable, especially when it comes to topics that have a societal theme, to talk to people about them. It’s important to hear perspectives that you may not agree with or that you haven’t considered […]. That way, you are challenged […] to reflect on your own opinions. Otherwise you can easily remain trapped in your own beliefs and echo chamber.’ – focus group 2, Ann (female).*

##### Forced to reflect on their own role as a medical student and future MD

Participants felt that the game including the reflection made them aware that issues related to climate change will be part of their future profession. Discussion of the roles and responsibilities of medical students and doctors in the field of climate change and health has served as a starting point for internal reflection.

‘*When I come to class as a medical student and start thinking about it, […] I believe that subconsciously, I will make a more active connection between [climate change and health]. […] For me, the small-group session has ensured that I am extra aware of the connection between the two, and also that as a future physician, it will be something I will encounter frequently and something I can actively engage with.’—focus group 1, Georgia (female).*

##### Realising that co-benefits of climate action for health provide tools for action

There was consensus that the co-benefits of climate action for health offer a strong argument for medical doctors to be involved in the fight against climate change. In addition to feelings of sadness and despair, participants were rather optimistic about the concept of co-benefits and the tools for action it provides. As Jake (male) put it: “*If we only heard a lecture on climate change and why we have to do something about it, I wouldn’t think it is my role [as a future doctor], however with the concept of co-benefits in mind, I think it is.”* In addition, participants indicated that medical doctors can contribute to climate change mitigation both at the individual patient level and at the system level. Some participants feel that an active societal and political role of medical doctors is becoming more common, and those participants wish to incorporate this in their future careers.

The focus group discussions revealed some ambivalence regarding whether doctors should take part in or refrain from climate activism. A neutral stance was mentioned by some to be important for maintaining a professional doctor-patient relationship. Not everyone agreed: ‘*I don’t think that encouraging people to be more aware of climate [change] is a political or climate activist choice or attitude. I think that it’s a fact that climate change impacts individual health as well as public health. Just as smoking […] in which doctors can also hold a strong opinion that you should not smoke, I also believe that doctors should be able to say […] that consuming red meat is not good for your own health, nor for the environment […]. I think it is important that doctors serve as role models in this regard. You don’t want a pulmonologist who smokes, and I wouldn’t want a doctor who consumes a lot of meat either.’—focus group 2, Beatrice (female).*

#### Strategies for improvement

The focus group participants were generally positive about the serious game. They mentioned a few aspects that could be improved.

##### Uniformity of teachers’ approach

Participants felt that uniformity of the teacher’s approach to facilitating the game and reflective discussion is important for ensuring equal learning outcomes across groups. For example, a slide deck to support the reflective discussion was used inconsistently.

*“A lot of the times there are educational sessions that are given by different teachers. […] Then the information you receive depends on which group you are in.” – focus group 2, Doug (male).*

##### Final exchange between groups to stimulate peer-learning

Since exchange between students with potentially different perspectives and attitudes is valued by participants, they suggested that the different smaller groups that played the game together could present their findings to each other at the end of the session. Participants believed this could be of added value, since other groups might have different opinions or gathered different insights (student, male): ‘*Explaining is one of the best ways to learn.’ – focus group 1, Jake (male).*

##### Integration of climate change and health education across the curriculum

Participants expressed their desire to have learned about climate change and health earlier in the curriculum. They feel the serious game will be more beneficial at an earlier time in their bachelor’s program. In addition, participants feel that the content of this serious game as a stand-alone topic is not enough and the topic should be integrated across subjects in the curriculum to be able to learn about the topic more in depth. They equally emphasized the significance of repetition for solidifying their knowledge. *Focus group 1: “ ‘I think it could be useful to know something about this topic earlier.’ ‘This timing is kind of late.’ ‘The overview of the material fitted nicely [in this course], but I think that these little pieces of information should be repeated more often.’” – a discussion between Karen (female) and Georgia (female).*

## Discussion

The primary objective of our study was to evaluate the knowledge and attitudes towards climate change and health of third-year bachelor medical students following a novel collaborative serious game. Our quantitative findings demonstrate a significant increase in self-reported knowledge. The results of the objective knowledge assessment were inconclusive. The tangible overview of complex interlinkages between climate change and health emerged as a crucial factor in developing students’ understanding.

Prior to playing the game, the majority of students acknowledged the relevance of climate change and health education to the medical curriculum and recognised their responsibility to mitigate the health sector’s environmental impact. Although their attitudes towards these topics showed a slight improvement after the game, the results did not reach statistical significance. Nevertheless, a significant shift towards more positive attitudes was noted regarding their role as future medical doctors in educating patients and society about the health impacts of climate change.

Playing the game as a medical student forced them to reflect on their own role and responsibilities. Learning about the co-benefits of climate action for health helped them realise that this concept provides tools for actions that they could implement in their future practice. It is important to acknowledge, however, that the increased awareness appeared to be accompanied by a greater level of concern about climate change and its impacts on health. Overall, the findings underscore that the serious game influences multiple domains of knowledge and attitude towards climate change and health among third-year bachelor medical students at Erasmus MC.

### Strengths and limitations

Our study presents the first evaluation of a novel educational methodology in the field of climate change and health. Our employment of a mixed-methods design enabled us to not only assess the impacts of the serious game, but also explore the underlying mechanisms responsible for its effects.

Nonetheless, participation in the serious game was voluntary, and the survey was completed by a mere 40.7% of the participating students. The low response rate may have occurred through several reasons, including student fatigue from frequent research requests and the impersonal approach to recruitment during the plenary introduction of the class. Moreover, the educational sessions took place during a very busy period for our students which might have decreased their willingness to participate in the study. Hence, selection and response bias might have been introduced in favour of those who already held relatively positive attitudes towards the subject. This assumption is in line with the predominantly positive attitude outlined in the pre-intervention survey responses. The sample size was relatively small, and since the number of focus groups was limited by the willingness of students to participate, data saturation could not be reached. Its insights might therefore lack a representative variety of perspectives. In addition, the moderators of the focus groups were part of the teaching staff for the serious game. Even though they did not teach the students who participated in the focus groups, this may have led the students to express more socially desirable views, although all efforts were made to ensure a safe and open environment for discussion. Furthermore, the absence of a control group in our study design hinders the attribution of any observed effect solely to the serious game. Another limitation is the use of predominantly self-assessed outcomes via a non-validated, nor pilot-tested survey. The validity of the objective knowledge assessment seemed particularly affected, which could explain its contradicting results. This underscores the importance of pilot-testing a newly developed questionnaire among the target population. Furthermore, assessment directly after the game may have directed students’ selective attention towards seeking out the right answer during class. Expanding the range of validated questionnaires used to evaluate climate change and health education, with the Sustainability Attitudes in Nursing Survey (SANS) as a notable example, is necessary for improving the quality of evidence [23]. Finally, the serious game was taught by thirteen different teachers. Although they all attended the same teacher training, their knowledge of the topic varied, as did their teaching skills and prior experience.

### Interpretation

Previous studies have confirmed that various approaches applied to climate change and health education or affiliated fields such as one health have a positive impact on the knowledge and attitudes of students. [24, 25, 26, 27, 28] Our study expands the existing evidence by adding a novel educational methodology to the palate of options for integrating climate change and health education into the medical curriculum.

Our qualitative analysis revealed that the serious game fostered a positive sense of responsibility. Although not statistically significant, survey responses also showed a trend toward increased feelings of responsibility for mitigating the environmental impact of healthcare. This effect can largely be attributed to the reflective component of the game. Using reflexive pedagogy in climate change and health education enables students to develop skills to reflect and address the complexities of the topic and advocate for change. [29] Likewise, the realisation that co-benefits of climate action for health provide tools for action was appreciated by the students. The participants of a similar study among undergraduate medical students appreciated the focus on action and the examples of concrete, achievable sustainability projects by their near peers [30].

While our students demonstrated a sense of responsibility to act within their roles as future doctors, the confrontation with the doomsday scenario being portrayed by the game appeared to elicit worries about climate change. Truly comprehending the size, severity and impact of the climate crisis can be inherently distressing. Worry therefore seems an inevitable consequence of climate change and health education. Nonetheless, climate-related worry or anxiety can paralyze individuals and lead to helplessness. More importantly, if individuals do not believe that they have the agency to act and bring about change, they may be overpowered by hopelessness, which may translate into inaction. [31] The focus group participants in this study, despite their feelings of responsibility as future doctors, expressed a similar sense of despair and distrust in the efficacy of their own, immediate, behavioural changes. Subsequently, the participants did not indicate taking immediate action in the weeks between the lesson and the focus groups.

One of the core objectives of overarching planetary health education is to equip students with the necessary values, skills and capabilities to sustainably promote human health [4]. Education in this field should therefore support emotional resilience and provide students with concrete tools for action, so that they feel a sense of agency to foster change [4, 32]. Consequently, dealing with diverse emotions elicited by the game becomes a crucial component of teacher’s professional development in this field [33].

**Fig. 4:**
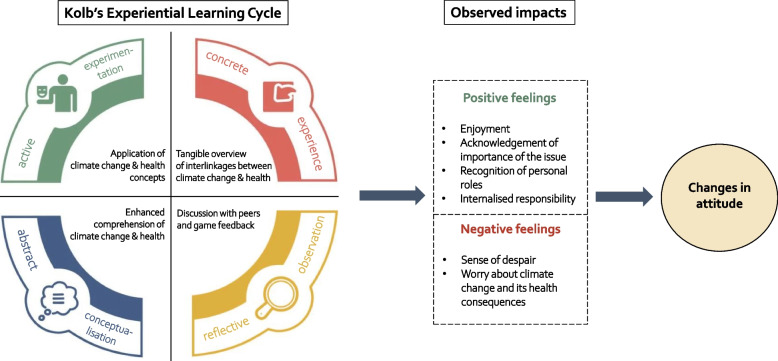
Outcomes of the serious game supported by Kolb’s Experiential Learning Theory [34]

Our serious game was collaborative, rather than competitive, and learning alongside peers with diverse opinions and attitudes was highlighted as influential in shaping students’ attitudes. In addition to collaborative engagement, serious games are considered effective at enhancing cognitive abilities and affective engagement and stimulating motivation to learn [11]. They foster a holistic understanding of scientific concepts, such as climate change and health, and prolong the retention of knowledge [11, 12]. The measured effects of our serious game on students’ attitudes may be explained through Kolb’s experiential learning theory (Fig. 4) [34]. According to this theory, changes in attitudes and behaviour are produced through experiential learning. This process unfolds in a circular manner, starting with concrete experiences of the topic during the game. These experiences prompt reflection, fostered by collaboration and game feedback, which in turn culminates in the comprehension of abstract ideas, specifically climate change and health. Ultimately, this iterative approach leads to active experimentation (test of the new concepts). It is assumed that the serious game provided students with a realistic and immersive (hence the previously mentioned ‘*wow-effect’*) overview of the interlinkages between climate change and health. This resulted in active engagement, assumed to generate both positive feelings of enjoyment and internalised responsibility but also negative feelings of, in this case, worry about the subject and its consequences.

### Recommendations

The findings of this study offer valuable insights into the effectiveness of a serious game for climate change and health education. Given the breadth of the topic, the intersection of personal opinions, social norms, and ideas on professional responsibilities, numerous aspects come into play, making it challenging to fully decipher all dynamics in a single study. Furthermore, longer-term evaluation studies are required to assess retention of knowledge, changes in attitudes over time and, ultimately, behavioural changes in students’ subsequent professional careers following climate change and health education.

Our key recommendations for educators include enhancing the integration of climate change and health education into the medical curriculum starting from the first year, and while doing so, considering the serious game as a potential tool for delivering complex concepts. Its content is applicable to all health-related settings across the globe, and the open questions being posed in Part 2 – Reflection Phase naturally lead to discussions about topics relevant to the setting in which the game is being played, including both low- and high-resource environments. At Erasmus MC, the serious game will be included as a mandatory component of the medical curriculum beginning in February 2024, and beginning in September 2024, the field of planetary health will be fully integrated into the new medical curriculum.

To ensure effective teaching in this emerging field, it is recommended that teachers are equipped to address such emotions in their class, by addressing them during the teacher training and encouraging teachers to reflect on their own emotional responses to the game. Moreover, it is important that the focus of Round 3 – Responsibilities of the reflection phase does not solely rest with individual lifestyle and choices, as this could evoke feelings of guilt. Furthermore, to counter worries with hope and the agency to foster change, a fundamental aspect of education on the topic remains encouraging students to contemplate potential actions they *can* undertake, whether individually or collectively within the health sector. It is important to acknowledge that meeting these recommendations may require more than a single two-hour session; instead, multiple educational sessions with reflective homework exercises might be warranted. Moreover, we plan to offer co-teaching opportunities with experienced teachers to address the variations in knowledge and expertise and ensure consistency in the teaching approach.

Future research should consider incorporating a control group, validated questionnaires, diverse settings and a follow-up measurement to evaluate attitude and retention of knowledge over time. Additionally, evaluation of mandatory educational sessions helps to recruit a larger, representative sample. This further improves the generalisability of the results and provides an opportunity to explore the differential effects among subgroups with specific demographic characteristics (e.g. age, gender, (parental) socioeconomic status).

## Conclusions

In conclusion, our novel collaborative serious game addressed an important gap in the Erasmus MC medical curriculum. While students held relatively positive attitudes towards the subject prior to playing the game, our study shows that the serious game influences multiple domains of knowledge and attitude regarding climate change and health. However, playing the game also raised worries related to the subject. Students indicate that the game can cultivate the necessary knowledge, attitudes and skills to promote health in times of a climate crisis through a tangible overview of the topic’s complexity, reflective engagement and interactive discussions with peers.

### Supplementary Information

Supplementary Material 1.

Supplementary Material 2.

Supplementary Material 3.

## Data Availability

The datasets used during the study are available upon reasonable request by contacting the corresponding author.

## Abbreviations

AMEE: Association for Medical Education in Europe
MC: Medical Center
MD: Medical Doctor
SANS: Sustainability Attitudes in Nursing Survey
SD: Standard deviation
