# Supplementary material for: Drawing a line from CO2 emissions to health—evaluation of medical students’ knowledge and attitudes towards climate change and health following a novel serious game: a mixed-methods study
Source: BMC Med Educ. 2024 Jun 5;24:626. doi: 10.1186/s12909-024-05619-4 (PMC11155108; doi:10.1186/s12909-024-05619-4)
Supplement: Supplementary file 1 — Supplementary Material 1. [file 12909_2024_5619_MOESM1_ESM.pdf]

Questionnaires evaluation Serious Game

Part 1a: pre-measurement

Q1: What is your age? *[free text]*

Q2: I am:
(male/female/non-binary/other/prefer not to say)

Part 1b: pre- and post-measurement

Q3: Do you think that climate change is caused by natural processes, human activity, or both?
□ Entirely by natural processes
□ Mainly by natural processes
□ About equally by natural processes and human activity
□ Mainly by human activity
□ Entirely by human activity

Q4: How worried are you about climate change?
□ not at all worried
□ not very worried
□ somewhat worried
□ very worried
□ extremely worried

To what extent do you agree with the following statements:

Q5: The impacts of climate change are a major issue for health care.
□ strongly agree
□ agree
□ neutral
□ disagree
□ strongly disagree

Q6: Education on this topic is important, because as a future medical doctor, I play an important role in informing **patients** about the health impacts of climate change.
□ strongly agree
□ agree
□ neutral
□ disagree
□ strongly disagree

Q7: Education on this topic is important, because, as a future medical doctor, I play an important role in informing **society** about the health impacts of climate change.
□ strongly agree
□ agree
□ neutral
□ disagree
□ strongly disagree

Q8: I feel that, once I am a medical doctor, I have **no** responsibility to reduce the environmental impact of health care.
□ strongly agree

 agree
 neutral
 disagree
 strongly disagree

Q9: Education about climate change and health has **no** place in the medical curriculum.
 strongly agree
 agree
 neutral
 disagree
 strongly disagree

Q10: I understand the effects of climate change on human health.
 strongly agree
 agree
 neutral
 disagree
 strongly disagree

Q11: I understand the ways in which the effects of climate change disproportionately affect vulnerable groups.
 strongly agree
 agree
 neutral
 disagree
 strongly disagree

Q12: I know what I can do tomorrow to reduce my own carbon footprint.
 strongly agree
 agree
 neutral
 disagree
 strongly disagree

Q13: Which of the following statements is or are true
 An average increase in temperature leads to aggravation of cardiovascular diseases, respiratory diseases, and an increase in infectious diseases; **(false)**
 Especially the elderly, homeless people, children, and chronically ill people are susceptible to the health consequence during heat waves; **(true)**
 Air pollution leads to more cardiovascular and respiratory diseases, and the effects worsen during heat waves; **(true)**
 An average higher temperature leads to increased plant allergenicity. **(false)**

Q14: Which of the following statements is or are true
 The United States of America, the South of Europe and the African continent are plagued by forest fires and are therefore the most severely affected by the climate crisis compared to other places in the world. **(false)**
 The (climate) impact of colonialism is recognized by climate science (the IPCC). **(true)**
 The Global North (including Russia, New Zealand, Australia, Japan, and Israel) is responsible for 85% of historical greenhouse gas emissions. **(false)**
 Climate change exacerbates gender inequality worldwide, as women are more dependent on agricultural production than men, among other reasons. **(true)**

Part 2: education evaluation (post-measurement)

Q15: I attended the lecture *Planetary Health* (part of Theme Ba3C)
☐ Yes, physically in the lecture hall
☐ Yes, online
☐ No

To what extent do you agree with the following statements?

Q16: The content of the serious game was well aligned with my prior knowledge.
☐ Completely agree
☐ Agree
☐ Neutral
☐ Disagree
☐ Completely disagree

Q17: The content of the serious game was relevant to my degree program.
☐ Completely agree
☐ Agree
☐ Neutral
☐ Disagree
☐ Completely disagree

Q18: I found the educational methodology (gamification) to be a pleasant way to cover this subject matter.
☐ Completely agree
☐ Agree
☐ Neutral
☐ Disagree
☐ Completely disagree

Q19: The serious game helped me to establish connections between climate change and its effects on disease and health.
☐ Completely agree
☐ Agree
☐ Neutral
☐ Disagree
☐ Completely disagree

Q20: How would you grade the serious game?

0 – 1 – 2 – 3 – 4 – 5 – 6 – 7 – 8 – 9 – 10

Q21: We would like to discuss this new skills education further with students during a one-time group interview. Compensation is available for participation.

If you are interested in participating, please indicate yes below and provide your email address in the next question.

☐ Yes
☐ No

Enter your email address: *[free text]*
