# Supplementary material for: Drawing a line from CO2 emissions to health—evaluation of medical students’ knowledge and attitudes towards climate change and health following a novel serious game: a mixed-methods study
Source: BMC Med Educ. 2024 Jun 5;24:626. doi: 10.1186/s12909-024-05619-4 (PMC11155108; doi:10.1186/s12909-024-05619-4)
Supplement: Supplementary file 2 — Supplementary Material 2. [file 12909_2024_5619_MOESM2_ESM.pdf]

Focus group protocol evaluation Serious Game

**Introduction**	• Oral consent<br>• Facilitators introduce themselves, emphasise neutrality<br>• Participants introduce themselves<br>• Aim of focus group and highlight definition of knowledge and attitude in this context<br>• Invite participants to share ideas, opinions and personal experiences, no right or wrong answers, no need to agree with each other<br>• Brief explanation of focus group set-up and role facilitations<br>• Invite participants to ask for explanation in case questions are unclear

**Section**	**Topic initiation question**	**Probing questions**
**Opening**	In pairs: Participants discuss the following question**: 'What are your thoughts about climate change? And how does the way you think about it influence your daily life?**	• What exactly leads you to think this way?<br>• How does this differ from your conversation partner?<br>• How do you notice that the way you think about [concerns/denial of climate change/disinterest] influences your daily life?
1: Introductory question	• Participants choose an association card (*Picture This!*) that, according to them, symbolises the issue of climate change and health.<br>• Participants introduce themselves one by one and explain their selected card. Try to understand what 'climate change and health' means to them.	
**Core questions**		
1: Playing card	• Display the Serious Game playing cards on a table. Ask each participant to choose a playing card that left a strong impression or provoked their thoughts during or after the game.<br>• *Participants explain their reasoning for their selected card to the group*. Try to uncover ***how*** the selected card provoked this impression or thought.<br>• Highlight notable differences among the selected cards to initiate a group discussion. Why	• Why or how did this particular card provoke your thoughts? Why is this important to you?<br>• Why this particular card, and not one of the other cards?

	do you think you’ve selected such different cards?	
2: Knowledge & attitude	• **What was your motivation for attending the serious game?**<br>• ***How* has the game, along with its reflection, influenced your understanding of climate change and health?**<br>• ***How* has the game, along with its reflection, influenced your attitude towards climate change and health?**	
3: Take-away	• **What was your main take-away after playing the game? How did the serious game guide you to this take-away message?**	• Can you describe a specific moment during the serious game that led you to this conclusion? Could you describe exactly how it occurred? What happened? To you or (in relation to) others?<br>• Can you describe your thoughts and emotions as you left the venue?
4: Professional role	• **How do you perceive the connection between what you have learned during the serious game and your (future) role as a healthcare professional?**	• Do you consider it relevant for your role as a (future) healthcare professional? Why (not)?
5: Strategies for improvement	• **What aspects of the serious game did you appreciate, and why?**<br>**How can we improve the serious game**? (*content, format, implementation*)	
6: Peer-to-peer interactions	• **How did you experience playing the game together with your group?**<br>• **How did playing the game collaboratively as a group influence your learning process?**<br>**What differences in knowledge and attitude regarding the topic existed within your group? How did you navigate these differences?**	
**Closing**	• Education on climate change and health has been offered in two formats: A lecture and a serious game. **Could you share how these two educational formats have impacted your knowledge and attitude? How do these impacts differ from each other, and what is the reason for these differences?**<br>• Facilitator summarises key issues that have been discussed.<br>• **Anything you would like to add to the summary?**<br>• Thank students for their participation.	
