# Supplementary material for: Drawing a line from CO2 emissions to health—evaluation of medical students’ knowledge and attitudes towards climate change and health following a novel serious game: a mixed-methods study
Source: BMC Med Educ. 2024 Jun 5;24:626. doi: 10.1186/s12909-024-05619-4 (PMC11155108; doi:10.1186/s12909-024-05619-4)
Supplement: Supplementary file 3 — Supplementary Material 3. [file 12909_2024_5619_MOESM3_ESM.pdf]

Quantitative results

Table 1: Knowledge before and after the serious game

**Objective knowledge**				
True/False questions	*Response*	Pre-measurement (n)	Post-measurement (n)	p-value[a]
(In)Direct health effects of climate change	*n (%) 0 or 1 correct*<br>*n (%) 2 correct*<br>*n (%) 3 or 4 correct*	4 (6.8%)<br>38 (64.4%)<br>17 (28.8%)	1 (1.7%)<br>53 (89.8%)<br>5 (8.5%)	0.023
Climate change vulnerabilities and inequity	*n (%) 0 or 1 correct*<br>*n (%) 2 correct*<br>*n (%) 3 or 4 correct*	34 (57.6%)<br>19 (32.2%)<br>6 (10.2%)	6 (10.2%)<br>25 (42.4%)<br>28 (47.5%)	**<0.001**
**Self-reported knowledge**				
Statement	*Response*	Pre-measurement (n)	Post-measurement (n)	p-value[a]
I understand the effects of climate change on human health.	*n (%) (strongly) disagree*<br>*n (%) neutral*<br>*n (%) (strongly) agree*	0<br>18 (30.5%)<br>41 (69.5%)	1 (1.7%)<br>1 (1.7%)<br>57 (96.6%)	**<0.001**
I understand the ways in which the effects of climate change disproportionately affect vulnerable groups.	*n (%) (strongly) disagree*<br>*n (%) neutral*<br>*n (%) (strongly) agree*	1 (1.7%)<br>5 (8.5%)<br>53 (89.8%)	1 (1.7%)<br>0<br>58 (98.3%)	**<0.001**
I know what I can do tomorrow to reduce my own carbon footprint.	*n (%) (strongly) disagree*<br>*n (%) neutral*<br>*n (%) (strongly) agree*	14 (23.7%)<br>14 (23.7%)<br>31 (52.5%)	1 (1.7%)<br>14 (23.7%)<br>44 (74.6%)	**<0.001**
[a] A significance level of $p < 0.004$ was maintained according to Bonferroni correction for multiple testing. P-values in bold are significant.				

Table 2: Attitude before and after the serious game

**Climate change worry and belief**				
Question/Statement	*Response*	Pre-measurement (n)	Post-measurement (n)	p-value[a]
Do you think that climate change is caused by natural processes, human activity, or both?	*n (%) entirely/mainly by natural processes*<br>*n (%) equally by natural processes and human activity*<br>*n (%) mainly/entirely by human activity*	1 (1.7%)<br>6 (10.2%)<br>52 (88.1%)	0<br>3 (5.1%)<br>56 (94.9%)	**0.003**
How worried are you	*% not at all worried/not very*	2 (3.4%)	0	**<0.001**

about climate change?	*worried*<br>*% somewhat worried*<br>*% very worried/extremely worried*	 32 (54.2%)<br>25 (42.4%)	 18 (30.5%)<br>41 (69.5%)	
The impacts of climate change are a major issue for health care.	*n (%) (strongly) disagree*<br>*n (%) neutral*<br>*n (%) (strongly) agree*	2 (3.4%)<br>4 (6.8%)<br>53 (89.8%)	4 (6.8%)<br>1 (1.7%)<br>54 (91.5%)	0.28
I feel that, once I am a medical doctor, I have **no** responsibility to reduce the environmental impact of health care	*n (%) (strongly) disagree*<br>*n (%) neutral*<br>*n (%) (strongly) agree*	48 (81.4%)<br>9 (15.3%)<br>2 (3.4%)	55 (93.2%)<br>3 (5.1%)<br>1 (1.7%)	0.014
Education on this topic is important, because as a future medical doctor, I play an important role in informing **patients** about the health impacts of climate change.	*n (%) (strongly) disagree*<br>*n (%) neutral*<br>*n (%) (strongly) agree*	5 (8.5%)<br>12 (20.3%)<br>42 (71.2%)	2 (3.4%)<br>2 (3.4%)<br>55 (93.2%)	**0.003**
Education on this topic is important, because as a future medical doctor, I play an important role in informing **society** about the health impacts of climate change.	*n (%) (strongly) disagree*<br>*n (%) neutral*<br>*n (%) (strongly) agree*	6 (10.2%)<br>16 (27.1%)<br>37 (62.7%)	2 (3.4%)<br>8 (13.6%)<br>49 (83.1%)	**0.003**
Education about climate change and health has **no** place in the medical curriculum.	*n (%) (strongly) disagree*<br>*n (%) neutral*<br>*n (%) (strongly) agree*	52 (88.1%)<br>6 (10.2%)<br>1 (1.7%)	57 (96.6%)<br>2 (3.4%)<br>0	0.005
[a] A significance level of $p < 0.004$ was maintained according to Bonferroni correction for multiple testing. P-values in bold are significant.				

Table 3: Post-intervention evaluation

**Question**	**Response**	**Total sample N = 72[a]**
The content of the serious game was well aligned with my prior knowledge.	*n (%) (strongly) disagree*<br>*n (%) neutral*<br>*n (%) (strongly) agree*	1 (1.4%)<br>2 (2.8%)<br>69 (95.8%)
The content of serious game was relevant to my degree program.	*n (%) (strongly) disagree*<br>*n (%) neutral*<br>*n (%) (strongly) agree*	0<br>4 (5.6%)<br>68 (94.4%)

I found the educational methodology (gamification) to be a pleasant way to cover this subject matter.	*n (%) (strongly) disagree*<br>*n (%) neutral*<br>*n (%) (strongly) agree*	1 (1.4%)<br>5 (6.9%)<br>66 (91.7%)
The serious game helped me to establish connections between climate change and its effects on disease and health.	*n (%) (strongly) disagree*<br>*n (%) neutral*<br>*n (%) (strongly) agree*	2 (2.8%)<br>1 (1.4%)<br>69 (95.8%)
How would you grade the skills education *Climate & Health Challenge*? (scale 0 - 10)	*Mean ± SD*	7.9 (0.9)
[a] The sample includes respondents who only completed the post-intervention questionnaire.		
